# Traumatic perforations of the tympanic membrane: immediate clinical recovery with the use of bacterial cellulose film^☆^

**DOI:** 10.1016/j.bjorl.2019.05.001

**Published:** 2019-06-11

**Authors:** Ana Mariana de Moraes Rebello Pinho, Carolina Christofani Sian Kencis, Dino Rafael Pérez Miranda, Osmar Mesquita de Sousa Neto

**Affiliations:** a Santa Casa de Misericordia de São Paulo, São Paulo, SP, Brazil; b Santa Casa de Misericordia de São Paulo, Faculdade de Ciencias Médicas, São Paulo, SP, Brazil

**Keywords:** Perforation of the tympanic membrane, Bacterial cellulose, Audiometry, Perfuração da membrana timpânica, Celulose bacteriana, Audiometria

## Abstract

**Introduction:**

Perforation of the tympanic membrane is a reasonably frequent diagnosis in otorhinolaryngologists’ offices. The expectant management is to wait for spontaneous healing, which usually occurs in almost all cases in a few weeks. However, while waiting for healing to be completed, the patients may experience uncomfortable symptoms. Although some research suggests the use of various materials to aid in the recovery of the tympanic membrane, none presented robust evidence of improvement in the cicatricial process. Nevertheless, the occlusion of the perforation with some material of specific texture and resistance can alleviate the patients’ symptoms and accelerate the healing process.

**Objective:**

To evaluate the clinical (symptomatic and functional) improvement after the placement of bacterial cellulose film (Bionext®) on tympanic membrane perforations (traumatic).

**Methods:**

We evaluated 24 patients, victims of traumatic perforations of the tympanic membrane, who were evaluated in the Otorhinolaryngology Emergency Room. Following otoscopy and audiometric examination was performed, before and after the use of cellulose film occluding the tympanic membrane perforation.

**Results:**

Twenty-four patients were included, whose degree of overall discomfort caused by the tympanic membrane perforation and the presence of symptoms of autophonia, ear fullness and tinnitus were investigated. The mean score attributed to the overall annoyance caused by tympanic membrane perforation was 7.79, decreasing to a mean value of 2.25 after the film application. Symptom evaluation also showed improvement after using the film: autophonia decreased from a mean value of 6.25 to 2.08, tinnitus from 7 to 1.92 and ear fullness from 7.29 to 1.96. The auditory analysis showed mean threshold values still within the normal range at low and medium frequencies, with slight hearing loss at acute frequencies, but with significant improvement at all frequencies, with the exception of 8000 Hz, after film use.

**Conclusion:**

The use of bacterial cellulose film fragment on traumatic perforations of the tympanic membrane promoted immediate functional and symptomatic recovery in the assessed patients.

## Resumo

**Introdução:**

A perfuração da membrana timpânica é uma condição clínica relativamente frequente em consultórios de otorrinolaringologistas. A conduta é quase sempre expectante, aguardando cicatrização espontânea, que costuma ocorrer na quase totalidade dos casos em algumas semanas. No entanto, enquanto não se completa, os pacientes mantêm sintomas desconfortáveis. Embora algumas pesquisas sugiram o uso de materiais diversos para auxiliar na recuperação da membrana timpânica, nenhuma apresentou evidência substancial de melhoria no processo cicatricial. Por outro lado, a oclusão da perfuração com alguns materiais de textura e resistência específicas poderia aliviar os sintomas dos pacientes durante o processo cicatricial.

**Objetivo:**

Avaliar a melhoria clínica (sintomática e funcional) após a colocação de película de celulose bacteriana (Bionext®) sobre a perfuração da membrana timpânica (traumática).

**Método:**

Foram avaliados pacientes com perfurações traumáticas da membrana timpânica que deram entrada no pronto-socorro de otorrinolaringologia. Eles foram avaliados em relação a suas queixas e exame audiométrico, antes e após a aplicação de película de celulose que oclui a perfuração da membrana timpânica.

**Resultados:**

Foram incluídos 24 pacientes, nos quais foram pesquisados o grau de incômodo global causado pela perfuração da membrana timpânica e a presença de sintomas de autofonia, plenitude auricular e tinnitus. A média da nota atribuída ao incômodo global causado pela perfuração foi de 7,79; caiu para valor médio de 2,25 após a aplicação da película. A avaliação dos sintomas também apresentou melhoria após uso da película, autofonia caiu de valor médio de 6,25 para 2,08; zumbido de 7 para 1,92 e plenitude auricular de 7,29 para 1,96. A análise auditiva apresentou um valor médio de limiares ainda dentro da normalidade em frequências baixas e médias, com perda de audição leve em frequências agudas, porém com melhoria significante em todas as frequências, com exceção de 8000 Hz, após a colocação da película.

**Conclusão:**

A aplicação de fragmento de película de celulose bacteriana sobre perfurações traumáticas da membrana timpânica promoveu recuperação funcional e sintomática imediata nos pacientes estudados.

## Introduction

Perforation of the tympanic membrane (TM) is a frequent occurrence today, most often caused by trauma. In cases of traumatic perforation, the main cause is associated with violence suffered by the patient caused by another person or by oneself, during the manipulation of sharp objects in the external auditory meatus.

As a consequence of this perforation, there may be hearing loss, usually of the conductive type, accompanied by symptoms such as ear fullness, tinnitus, autophony and middle ear infections.^1^ The hearing loss perceived by the patient becomes more intense the greater the perforation is, being worse at lower frequencies.^2^

Traumatic perforation of the TM can heal spontaneously in 70–90% of cases, but the duration of the process until the complete closure may be long and sometimes take one month or longer.^3^ Surgical management, such as tympanoplasty or myringoplasty to close the tympanic perforation is more invasive and costly and, therefore, is an option reserved for cases in which there was no spontaneous resolution of the condition. Thus, for these reasons, the primary treatment is a conservative one.^4^, ^5^

The process of tympanic membrane healing occurs through the migratory proliferation of the keratinized squamous epithelium, its outermost layer, which advances into the connective tissue, and the permanence of the perforation occurs when this growth continues into the border of its margin, toward the middle ear.^6^ In order to accelerate the closure of this perforation, several techniques have been employed, and numerous studies have used biological materials as support or grafts.

The earliest attempts to close the tympanic membrane with heterologous materials appeared in the seventeenth century. Banzer in 1640 used a pig bladder fragment for this purpose. In addition, Toynbee applied a rubber-like substance in 1853, while Blake used a paper disk in 1887.^7^, ^8^ Since then, several other materials have been employed in the search for an ideal material.

The paper graft, introduced by Blake, is still the most widely used material. Camnitz and Bost in 1985^9^ applied the paper patch under topical anesthesia in 50 patients, demonstrating the efficacy of this method, with recovery rates of 92% and healing in 2–3 weeks. The use of biological materials has also been employed. Sayin et al.^10^ used a graft of hyaluronic acid ester (Epifilm®) and compared it with spontaneous healing. A total of 155 patients were followed for months and, although there was no significant difference between the efficiency of the two methods, the use of a hyaluronic acid patch showed a faster tympanic membrane closure process.

The vast majority of studies have not shown a significant difference between these new techniques and the process of spontaneous healing in relation to faster or more frequent recovery. However, one important observation is that such attempts did not show any harm to the healing process or tympanic membrane.^11^

Considering the expectant behavior in the management of traumatic perforation of the TM and the persistence of patients’ uncomfortable symptoms until complete healing of the TM, this study proposes the placement of a bacterial cellulose film (Bionext®) fragment on the tympanic damaged area, aiming at offering immediate functional recovery and thus promote symptom and functional relief. Whereas most literature studies describe the placement of autologous or heterologous material with the objective of evaluating the healing velocity of the tympanic membrane, this study has the advantage of proposing the evaluation of clinical improvement (symptomatic and functional) after the placement of Bionext® film fragment.

## Cellulose film – Bionext®

Bionext® film originates from the fermentation of the *Acetobacter xylinum* bacteria. The cellulose synthesized by these bacteria may present as a flexible, semitransparent film or a gelatinous sheet, approximately 0.5-cm thick. After its processing, it contains no additives, being pure cellulose consisting of biodegradable, non-toxic, non-pyrogenic, and sterile polysaccharides. It is an inert substance, very resistant and insoluble in all the organic solvents; it has specific physical characteristics, such as: defined permeability to fluids and gases, tensile and elongation strength. It is currently used as a temporary skin graft and is useful for the dressing of burn injuries, dermabrasion or skin donor areas.^12^, ^13^, ^14^ It has also been used as a substitute for meninges, as a coating material for intravascular stents and varicose ulcers of limbs.^15^

Kato et al.^16^ evaluated the response of the TM epithelium and tympanic cavity mucosa of guinea pigs experimentally submitted to traumatic perforation after receiving implants of the cellulose film produced by the *Acetobacter xylinum* bacteria, showing that the healing process when using this material occurs in a similar way to the spontaneous healing, without any tissue damage, including functional recovery assessed by otoacoustic emissions. They concluded that the histological parameters of acute inflammation (neutrophils, fibrin and vascular neoformation) and chronic inflammation (fibroblasts, mononuclear cells – lymphocytes and macrophages), in general, behave similarly in the presence or not of the cellulose film. This behavior is also observed in the long-term evaluation.

Considering the conclusion of the study by Kato et al.^16^ and several other studies that have already used this material in guinea pigs or humans, it can be observed that it poses minimal risk, as it is a biological and inert material. As an observation, there may be an inflammatory allergic reaction if the patient is allergic to the cellulose compound of which Bionext® is made.

## Material and methods

The study is a prospective clinical trial, which included patients with traumatic perforations of the tympanic membrane, over 18 years of age, who were evaluated in the Otorhinolaryngology Emergency Department between May 2015 and September 2016. The study was approved in the Ethics Committee of the institution Santa Casa de Misericórdia de São Paulo under opinion number 912.218 – 12/14/2014.

Patients who had either otorrhea or were unable to give a relevant history at the first consultation or to collaborate with the physical examination and/or the bacterial cellulose film placement procedure on the traumatized TM were excluded from the study.

Once the inclusion criteria had been met, patients answered a questionnaire in which they graded the intensity of overall annoyance caused by the condition, using a scale from 0 (zero) to 10 (ten), with zero being no annoyance and 10 the maximum degree of annoyance. They also reported whether they noted tinnitus, autophonia or ear fullness, and classified these symptoms by the degree of annoyance, using the same scale from 0 to 10. Then the patient underwent a tonal and vocal audiometry test to identify auditory thresholds, which could characterize hearing impairment.

The bacterial cellulose film fragment was then placed over the tympanic perforation, in a lateral position to the TM, under direct visualization with the aid of a surgical microscope. The DF Vasconcelos microscope, model M900, was used, and the magnification varied according to the need and the comfort of the examiner. The design of this cellulose fragment was carried out with a calculated size to be slightly larger than the area of the perforation so as to completely cover it and remain supported by the remaining TM. For greater adhesion to the outer layer of the TM, a small amount (approximately 1–2 drops) of saline solution, was placed directly on the bacterial cellulose film. The procedure was performed without the need for local anesthesia and was well tolerated by all patients.

**Figure 1:**
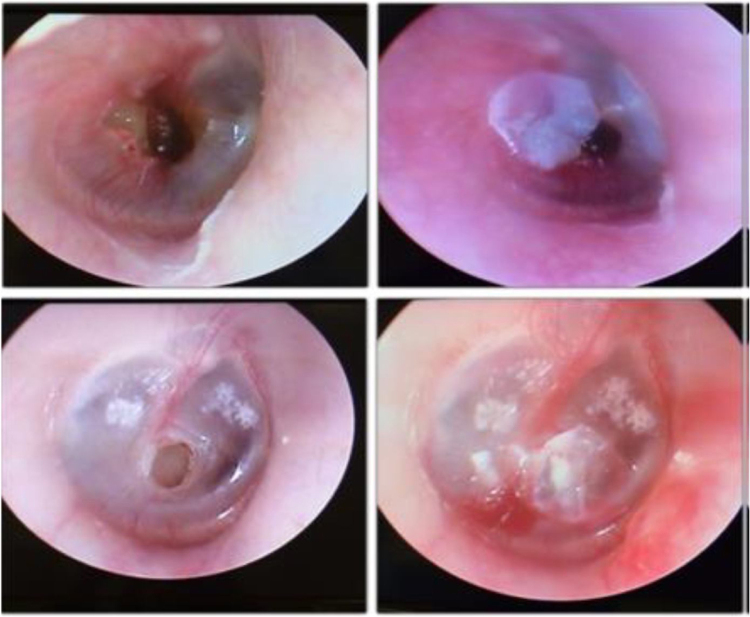
Patients’ otoscopy procedures: on the left showing the perforation and on the right, after the cellulose film placement.

Once the Bionext® film fragment was placed, the same questionnaire questions were repeated, and a new tonal and vocal audiometry test was performed. Thus, we obtained elements to objectively and subjectively evaluate whether the clinical (symptomatic and functional) recovery of the patient occurred, with the patients being their own controls. Fig. 1 depicts the video-otoscopy image of two patients before and after the placement of the Bionext® film.

All patients who underwent Bionext® placement had a return consultation scheduled for 30 days after the first consultation for the reassessment of otoscopy and clinical status, reporting whether the auditory symptoms persisted in relation to the last consultation. During this period they were advised to protect the affected ear from contact with water, being also prohibited from immersion in a swimming pool or the sea. The patients were advised to return at any moment if there was any complaint associated with the affected ear.

The Wilcoxon test was used for non-parametric variables and Pearson's correlation coefficient for comparison between quantitative variables.

## Results

Overall, 24 patients met the inclusion criteria and were selected for the study. Among them, 45.8% (*n* = 11) were males and 54.2% (*n* = 13) females. The age ranged from 19 to 58 years, with a mean of 34 ± 10.4 years. Table 1 summarizes the main epidemiological data of patients that met the inclusion criteria. It also shows data on the time between the trauma and the first consultation, the side affected by the perforation and the area of the tympanic membrane that was affected by the perforation.

**Table 1:** Epidemiological data.

*Gender*	
Male	11 (45.8%)
Female	13 (54.2%)

*Affected side*	
Right	6 (25%)
Left	18 (75%)
*Age*	34 ± 10.4 years old
Elapsed time	8.6 ± 4.9 days
Perforation Area	25% ± 14%

Regarding the etiology, the most common was violence, in 62.5% (*n* = 15) of the cases, which included victims of aggression such as punches and slaps, and perforations caused by sports injuries. The second most common cause was the introduction of a pointed object into the external auditory meatus, in 33.3% (*n* = 8) of the cases, and finally, only one patient (4.5%) had a perforation due to an abrupt Valsalva maneuver, while attempting to prevent a sneeze.

**Figure 2:**
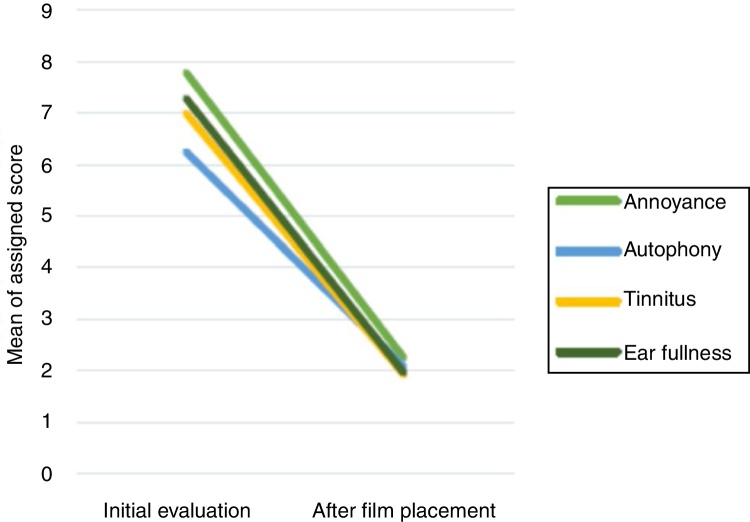
Comparison between the mean of the initial values and after the film placement.

The questionnaire data, related to the scoring of the symptoms in a scale from 0 to 10, are summarized in Table 2. It shows the mean values for each complaint at the time of the first evaluation and after the cellulose film fragment placement. All patients reported ear fullness and tinnitus, while autophony was present in 91% (*n* = 22). All reported a decrease in the annoyance after the film placement, with significant difference for all analyzed variables. Fig. 2 shows the comparison between the values at the first and the second moments for each symptom.

**Table 2:** Questionnaire data – mean scores assigned to each complaint.

	Overall annoyance	Autophony	Tinnitus	Ear fullness
Initial evaluation	7.79 ± 2.6	6.25 ± 3.1	7 ± 3.37	7.29 ± 1.96
After film placement	2.25 ± 2.1	2.08 ± 2.2	1.92 ± 1.97	1.96 ± 2.03
*p*-Value	<0.001	<0.001	<0.001	<0.001

Wilcoxon test.

**Figure 3:**
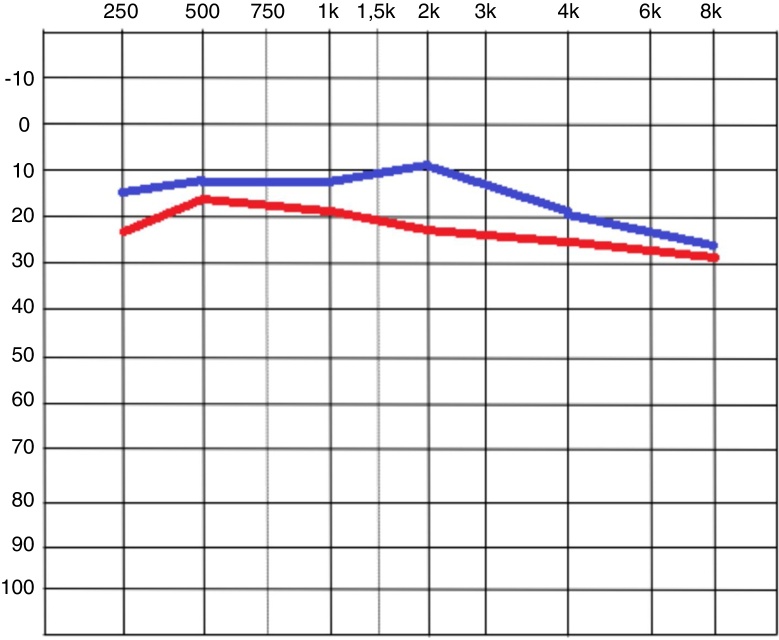
Tonal audiometry image, comparing the means of the initial values in red, and after the film placement, in blue.

Table 3 shows the mean values found for each frequency in the tonal audiometry, before and after the cellulose film placement, while Fig. 3 shows this exam at both moments. The difference between the values showed a significant improvement (*p* < 0.005) for all frequencies, except for the 8000 Hz frequency (*p* = 0.124).

**Table 3:** Mean values for each tone threshold obtained at the time of the first evaluation and after film placement.

	Initial moment	After film placement	*p*-Value
250 Hz	23.13 dBHL ± 12.92	15.21 dBHL ± 8.6	<0.001
500 Hz	18.54 dBHL ± 10.05	12.08 dBHL ± 7.21	<0.001
1000 Hz	19.38 dBHL ± 9.36	12.08 dBHL ± 8.57	0.001
2000 Hz	23.33 dBHL ± 16.8	9.65 dBHL ± 18.59	0.001
4000 Hz	24.79 dBHL ± 20	18.54 dBHL ± 21.23	<0.001
8000 Hz	27.50 dBHL ± 19.9	25 dBHL ± 20.43	0.124

Wilcoxon test.

The vocal audiometry included only the Speech Recognition Threshold (SRT) analysis. The initial SRT was 21.25 dBHL ± 8.50, whereas it was 13.13 dBHL ± 7.49 after the film placement. This difference was also significant (*p* < 0.005).

We investigated whether there was any association between the perforation area and the score attributed to each complaint, and no correlation was found. Similarly, the size of the perforation area and the magnitude of the improvement demonstrated by the patient (the difference between the first and second moments) were compared, and no significant relationship was identified. This comparison was performed using Pearson's correlation coefficient.

Only 50% (*n* = 12) of the patients came back for the scheduled return consultation after one month, and of these, 16.6% (*n* = 2) did not have a fully healed tympanic membrane yet. These two patients showed, at the moment of the follow-up consultation, the presence of granulation tissue next to cellulose film and otorrhea and were treated with topical antibiotic therapy. A new return was scheduled for two months later, when all returning patients were asymptomatic.

## Discussion

Traumatic perforation of the tympanic membrane is known to have a good prognosis and spontaneous recovery, even without treatment.^2^ However, while awaiting the healing of the membrane, the patient has to live with the symptoms caused by the perforation. Several studies have been carried out with the objective of comparing the healing velocity of TM in experiments using different techniques and materials, however, none of them assessed the patient's degree of annoyance caused by the hearing loss or tinnitus. The aim of these studies was not to determine or characterize the annoyance experienced by patients.

The use of bacterial cellulose film (Bionext®) in patients, covering the traumatic perforation of the tympanic membrane, seems to be an alternative treatment to minimize this annoyance; it has shown to be quite effective in reducing symptoms.

All the evaluated symptoms occurred with significant frequency and intensity, since symptoms showed mean scores always close to 7; after the cellulose film placement, the improvement of the score given to each symptom was significant, decreasing to mean values close to 2. This improvement confirms the efficacy of this method to relieve the annoyance caused by perforation.

This improvement of the subjective parameters, assessed by the symptom scores, was confirmed by the measurement of the tonal and word recognition thresholds. Although these audiometric procedures can also be considered subjective, their degree of subjectivity is lower.

The auditory assessment showed initial thresholds ranging from 18.54 to 23.33 dBHL for low and medium frequencies (250 Hz, 500 Hz, 1000 Hz and 2000 Hz), and 24.79–27.50 dBHL for high frequencies (4000 Hz and 8000 Hz). Although the expected result in cases of tympanic membrane perforation is a more intense loss of conduction at low frequencies,^2^, ^8^ our evaluation of tonal thresholds showed a greater loss at high frequencies. One possible explanation is that such patients have suffered, in addition to the tympanic perforation, an acoustic trauma, which could explain the alteration at the base of the cochlea.

After the film placement, an immediate and significant improvement in the tonal thresholds was observed for all frequencies, except for 8000 Hz, showing a recovery of the TM function. This result is similar to that found in the literature. Spandow^17^ and Silverstein^8^ also evaluated the presence of auditory threshold alteration after paper patch placement. Both found marked improvement at low and medium frequencies without significant improvement at high frequencies (4000 Hz).

Some variables could be determinant and interfere with the results related to symptoms and audiometry, such as the size of the perforation.

This study sample work was relatively homogeneous, with perforations that affected on average 25% of the TM, with no case of perforation greater than 50%. As observed, both the symptoms and their improvement are unrelated to the size of the perforation, and treatment with the film application for any perforation, regardless of its size, is indicated. The annoyance caused by these perforations seems to be more related to the simple presence of TM discontinuity and not to a more intense impairment of the middle ear amplification mechanisms (in this case, catenary and hydraulic).

During the study period we had a considerable number of patients who failed to return, but this fact did not interfere with the final results, since our objective was to evaluate the immediate response to the film placement and not its evolution in the medium or long term. Despite this failure, our experience showed that the procedure is safe, as only two cases had complications (humidity and granulations in the tympanic membrane), which were easily controlled with a further 30-day follow-up. It can be assumed that the characteristics of the cellulose film, such as its thickness, malleability and permeability, were adequate for the proposed objectives and contributed to the patients’ clinical recovery.

Generally, the application of bacterial cellulose film has shown to be an innocuous procedure, with a low complication rate, well tolerated by the patient and with a clear improvement of the auditory symptoms and thresholds, being therefore a good alternative for the clinical treatment while waiting for the tympanic membrane to heal.

## Conclusion

The use of a bacterial cellulose film fragment on traumatic perforations of the tympanic membrane promoted immediate functional and symptomatic recovery in the assessed patients.

## Conflicts of interest

The authors declare no conflicts of interest.
